# Latvian herbal medicines under the infrared lens: An FTIR-ATR dataset

**DOI:** 10.1016/j.dib.2025.112378

**Published:** 2025-12-11

**Authors:** Una Lote Vītoliņa, Jurģis Krūmiņš, Renāte Teterovska, Ance Bārzdiņa, Baiba Mauriņa, Dace Bandere, Agnese Brangule

**Affiliations:** a Department of Applied Pharmacy, Riga Stradiņš University, LV-1007 Riga, Latvia; b Baltic Biomaterials Centre of Excellence, Headquarters at Riga Technical University, LV-1658 Riga, Latvia; c Department of Pharmacology and Pharmacotherapy, Riga Stradiņš University, LV-1007 Riga, Latvia

**Keywords:** Fingerprinting, Chemical profiling, Plant identification, Medicinal plants, Plant characterization

## Abstract

As more individuals use plant-derived products as prophylactic and therapeutic remedies, the herbal medicine market continues to grow rapidly. This trend shows the need for more advanced analytical methods, such as spectral “fingerprinting” – creating spectrum with a distinctive pattern, which is further used as a unique identifier for a plant.

The dataset was generated using Fourier-transform infrared spectroscopy with attenuated total reflectance (FTIR-ATR) to produce distinctive spectral fingerprints of Latvian medicinal plants: red and white clover, purple coneflower, cowslip, common jasmine, sandy everlasting, horse chestnut, pot marigold, chamomile, coltsfoot, common daisy, elderberry, cornflower, Jerusalem artichoke, fireweed, common and Midland hawthorn, small-leaved and large-leaved lime, yarrow, and meadowsweet.

The samples were obtained from ten Latvian herbal medicine producers, along with one additional sample collected independently by Riga Stradiņš University researchers. The analytical method requires minimal sample preparation, involving only the grinding of medicinal plants into fine powder.

The research yielded 329 spectra, each corresponding to a distinct species of medicinal plant, which are sorted in 19 CSV files. These files contain spectral data suitable for various applications, including chemical characterization, species classification, and quality control. In addition, the dataset can be used for multivariate data analysis or chemometrics and will allow for signal processing and spectroscopic data handling.

Specifications Table

**Table:** 

Subject	Health Sciences, Medical Sciences & Pharmacology
Specific subject area	The study applies a non-destructive method, FTIR-ATR Spectroscopy, to obtain fingerprint spectra of Latvian medicinal plants.
Type of data	Pre-processed Table (.csv format)
Data collection	The dataset consists of 19 files (.csv format) containing 329 FTIR-ATR spectra and one table (.csv format) providing a summary of the names of medicinal plants in English, Latin, and Latvian. Herbal samples were selected from ten different Latvian producers, and one sample set was collected by researchers. FTIR-ATR spectra were taken directly with Thermo Fisher Scientific Nicolet iS5 with a diamond crystal, recorded from 500 to 3800 cm^−1^ with 16 scans and a resolution of 4 cm^−1^, optical velocity of 0.4747, and aperture of 100 %. Each plant sample has at least four replications. Baseline correction and normalization were done in SpectraGryph 1.2.16.1 [1] to the most intense band in 850–1850 cm⁻¹.
Data source location	Country: Latvia Affiliation: Riga Stradins University, Faculty of Pharmacy, LV-1007, Riga, Latvia
Data accessibility	Repository name: Zenodo Data identification number: 10.5281/zenodo.16892975 [2] Direct URL to data: https://doi.org/10.5281/zenodo.16892975
Related research article	None

## 1 Value of the Data

• **Direct usability across software environments and training set development for machine learning**
  FTIR-ATR spectra are provided in CSV format, making them directly compatible with statistical and chemometric software (e.g., ORIGIN, SIMCA). These data are well-suited for building training sets in machine learning applications such as plant authentication, classification, and quality control, thereby reducing the need for extensive new data collection.
• **Chemical fingerprinting for herbal medicine characterization**
  The spectra can serve as chemical fingerprints for herbal medicine characterization, and combined with other analytical methods, such as high-performance liquid chromatography (HPLC), and thin-layer chromatography (TLC), they provide a more comprehensive view of the plant’s chemical composition, which can be used to confirm the herbal medicines authenticity and formulation.
• **Non-destructive analysis enabling sample preservation**
  As a non-destructive technique, FTIR-ATR offers a broad overview of plant chemical composition, requiring only a small sample amount while leaving the material unaltered. This is important because the samples remain available for further analysis, verification, or future use.
• **Applications in phytochemical profiling**
  The dataset supports advanced phytochemical profiling and authenticity assessment in herbal-derived products. Representing medicinal plants from Latvia, it provides a basis for comparing chemical profiles with those from other regions, as plant composition can vary with species, growth location, age, harvesting season, drying conditions, and other factors. This collection can therefore serve as a valuable reference database for comparative analysis.

## 2 Background

The use of herbal medicine continues to grow across Europe – the market was worth USD 65 billion in 2024 and is projected to reach USD 110 billion by 2033 [3]. This trend is driven by a combination of historical traditions and modern consumer preferences.

Despite their natural origin, herbal medicines can pose higher risks of adverse effects when contaminated or sourced from unknown origins [[4], [5], [6]]. With market growth, counterfeit and impure herbal medicines are increasingly prevalent [[7], [8], [9]]. For example, a study of herbal products sold for addiction treatment in Iran 74 % contained illegal substances [10].

The World Health Organization, the Food and Drug Administration of the USA, and the European Medicines Agency have approved spectral fingerprinting as a valid method for herbal medicine quality screening [[11], [12], [13]]. With large amounts of data, correlations between specific species can be found and the entire FTIR-ATR spectrum can be used as a fingerprint [14]. Moreover, combining FTIR-ATR with HPLC and TLC, a more comprehensive view can be given about the medicinal plants' chemical composition [15].

The selected medicinal herbs are well-known in traditional Latvian folk medicine for their therapeutic properties and are still used to this day [16].

## 3 Data Description

The dataset consists of 19 CSV files containing 329 FTIR-ATR spectra of Latvian medicinal plants, acquired in the spectral range of 500–3800 cm⁻¹.

For this study, products from ten different Latvian herbal medicine producers were selected, including two biologically certified producers (Producer no 2 and no 7). In addition, one sample set was collected directly by researchers to serve as a reference from a natural source.

The dried tea samples were purchased from reputable Latvian herbal medicine producers who collected the plants in 2019–2020 at their recommended harvest age according to the European Pharmacopoeia [17].

Table 1 provides a summary of the names of medicinal plants in English, Latin, and Latvian and part used.

**Table 1:** List of medicinal plant species in Latin, English, and Latvian and part used.

Part used	Latin	English	Latvian
Flower	*Achillea millefolium* L.	Yarrow	Parastais pelašķis
	*Aesculus hippocastanum* L.	Horse chestnut	Parastā zirgkastaņa
	*Bellis perennis* L.	Common daisy	Ilggadīgā mārpuķīte
	*Calendula officinalis* L.	Pot marigold	Ārstniecības kliņģerīte
	*Centaurea cyanus* L.	Cornflower	Zilā rudzupuķe
	*Chamaenerion angustifolium* (L.) Schur	Fireweed	Šaurlapu ugunspuķe
	*Echinacea purpurea* L.	Purple coneflower	Purpursarkanā ehinācija
	*Helianthus tuberosus* L.	Jerusalem artichoke	Bumbuļu topinambūrs
	*Helicrysum arenarium* Moench	Sandy everlasting	Dzeltenā salmene (kaķpēdiņa)
	*Jasminum officinale* L.	Common jasmine	Jasmīns
	*Matricaria recutita* L. (*syn. M. chamomilla* L.)	Chamomile	Smaržīgā (ārstniecības) kumelīte
	*Primula veris* L. (*syn. P. officinalis* Hill)	Cowslip	Gaiļbiksīte
	*Sambucus nigra* L.	Elderberry	Melnais plūškoks
	*Tilia cordata* Mill. (*syn. T. parvifolia* Ehrh.)	Small-leaved lime (linden)	Parastā (ziemas) liepa
	*Tilia platyphyllos* Scop.	Large-leaved lime (linden)	Platlapu (vasaras) liepa
	*Trifolium pratense* L.	Red clover	Sarkanais āboliņš
	*Trifolium repens* L.	White clover	Baltais āboliņš
Fruit	*Crataegus laevigata* (Poir.) DC. (*syn. C. oxyacantha* L.)	Midland hawthorn	Nogludinātā (divirbuļu) vilkābele (krustābele)
	*Crataegus monogyna* Jacq.	Common hawthorn	Vienirbuļa vilkābele (krustābele)
Leaf	*Tussilago farfara* L.	Coltsfoot	Māllēpe
Flower, leaf, stem	*Filipendula ulmaria* (L.) Maxim. (*syn. Spiraea ulmaria* L.)	Meadowsweet	Parastā vīgrieze

File names follow standardized format: Plant name (in both English and Latin)_Producer_Measurement replicate number. For example, White clover_Trifolium repens_Pro1_1. Each producer is assigned with a unique identifier, such as Pro1, Pro2, and so on. All file names and their number of replicates and total samples are listed in Table 2.

**Table 2:** List of CSV file names, producers, number of replications and total samples.

	File name	Producers	Minimal number of replicates	Number of spectra for the medicinal plant
1.	CSV_FTIR_ATR_Red clover_Trifolium pratense	Pro1, Pro2	5	11
2.	CSV_FTIR_ATR_White clover_Trifolium repens	Pro1, Pro2	5	11
3.	CSV_FTIR_ATR_Purple coneflower_Echinacea purpurea	Pro2, Pro3	5	11
4.	CSV_FTIR_ATR_Cowslip_Primula veris	Pro1, Pro2, Pro3	5	15
5.	CSV_FTIR_ATR_Common jasmine_Jasminum officinale	Pro1	5	5
6.	CVS_FTIR_ATR_Sandy everlasting_Helicrysum arenarium	Pro3, Pro4	5	10
7.	CSV_FTIR_ATR_Horse chestnut_Aesculus hippocastanum	Pro1, Pro2	5	10
8.	CSV_FTIR_ATR_Pot marigold_Calendula officinalis	Pro1, Pro3, Pro4, Pro5	5	20
9.	CSV_FTIR_ATR_Chamomile_Matricaria recutita	Pro1, Pro2, Pro3, Pro4, Pro6, Pro7, Pro8	5	40
10.	CSV_FTIR_ATR_Coltsfoot_Tussilago farfara	Pro1, Pro3	5	10
11.	CSV_FTIR_ATR_Common daisy_Bellis perennis	Pro2	5	5
12.	CSV_FTIR_ATR_Elderberry_Sambucus nigra	Pro1	5	5
13.	CSV_FTIR_ATR_Cornflower_Centaurea cyanus	Pro1, Pro2, Pro9	5	16
14.	CSV_FTIR_ATR_Jerusalem artichoke_Helianthus tuberosus	Pro1, Pro2	5	10
15.	CSV_FTIR_ATR_Fireweed_Chamaenerion angustifolium	Pro1, Pro2, Pro3	5	15
16.	CSV_FTIR_ATR_Hawthorn fruit_Fructus Crataegi	Pro1	5	5
17.	CSV_FTIR_ATR_Linden flower_Flos Tiliae	Pro1, Pro2, Pro3, Pro4	5	22
18.	CSV_FTIR_ATR_Yarrow_Achillea millefolium	Pro1, Pro2, Pro3, Pro10, Pro11.1, Pro11.2	4	45
19.	CSV_FTIR_ATR_ Meadowsweet_Filipendula ulmaria	Pro1, Pro2, Pro3; Pro11.1, Pro11.3, Pro11.4	5	63

Table 3 shows the approximate geographical coordinates of the medicinal plant collection sites.

**Table 3:** Geographical coordinates of the producers harvest sites.

Producers	Region in Latvia; Approximate coordinates WGS84
Pro1	Vidzeme, 57.03° N, 25.61° E,
Pro2	Vidzeme, 56.89° N, 26.29° E
Pro3	Zemgale, 56.60° N, 23.30° E
Pro4	Producer has not specified
Pro5	Vidzeme, 57.31° N, 25.35° E
Pro6	Vidzeme, 57.31 ° N, 25.27° E
Pro7	Vidzeme, 57.31° N, 25.35° E
Pro8	Zemgale, 56.81° N, 24.25° E
Pro9	Vidzeme, 56.86 ° N, 24.23° E
Pro10	Producer has not specified
Pro11	Vidzeme, 57.01° N, 24.55° E

**Fig. 1:**
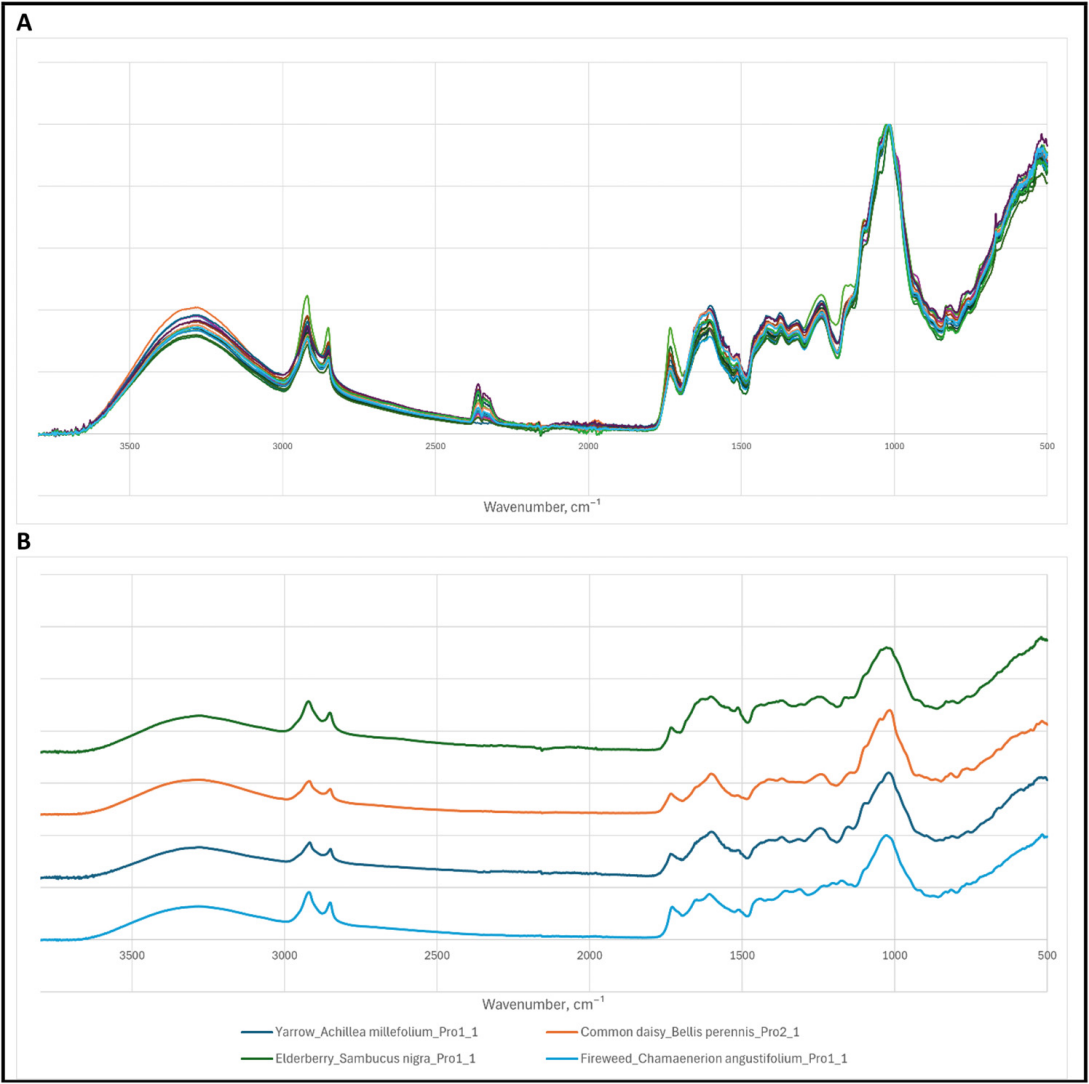
FTIR-ATR spectra of medicinal plants: (A) Cornflower (Centaurea cyanus) with all the samples from Producer no 1, 2, and 9; (B) Yarrow (Achillea millefolium), Elderberry (Sambucus nigra) and Fireweed (Chamaenerion angustigolium) samples from Producer no.1 and Common daisy (Bellis perennis) sample from Producer no.2.

As shown in Fig. 1, the FTIR-ATR full spectra of a single medicinal herb are grouped into one file, which includes only the producers from whom researchers have collected the sample.

**Fig. 2:**
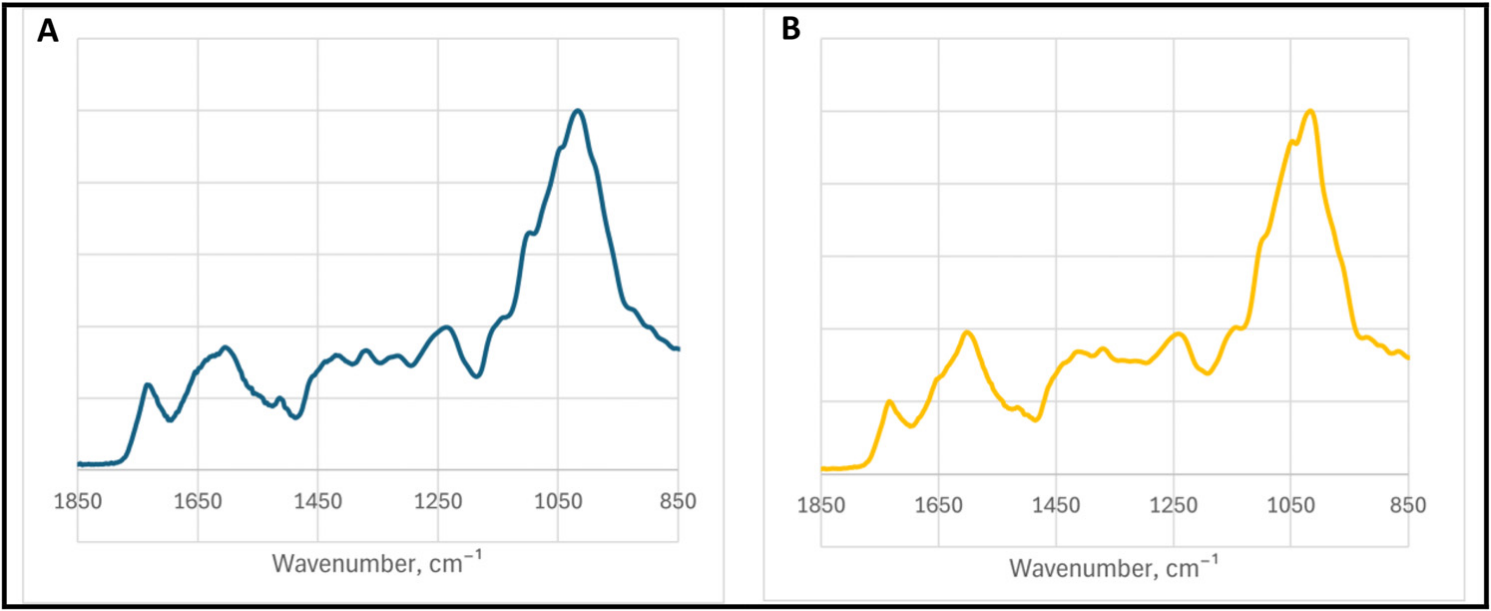
Fingerprint region of medicinal plant FTIR-ATR spectra: (A) Cornflower (Centaurea cyanus) Producer no.1, sample no.1; (B) Common daisy (Bellis perennis) Producer no.2, sample no 1.

The fingerprint region is one of the most important parts of the spectrum. The most intense signals are observed between 850 and 1850 cm^−1^, as shown in Fig. 2.

## 4 Experimental Design, Materials and Methods

### 4.1 Sample collection and preparation

Each producer (Pro1–Pro11) is associated with a specific geographical region in Latvia, and the approximate coordinates (WGS84) of these regions are provided in Table 3.

Herbal samples were selected from ten different Latvian producers, including two biologically certified producers (producer no 2 and no 7) in a form of dried tea; in addition, one sample was collected by the researchers and dried under varying conditions: 35 °C, 58 °C, 70 °C, and ambient room temperature.

Dried tea samples were purchased from reputable Latvian herbal medicine producers, with plants collected in 2019–2020 at the harvest age specified by the European Pharmacopoeia [17].

All dried plants were ground into a powder and sifted through a 2 mm sieve. The resulting powders were stored at room temperature for further analysis.

### 4.2 Instrument setup and spectral data acquisition

Attenuated total reflection (ATR) spectroscopy spectra of all herbal samples were taken with Thermo Fisher Scientific Nicolet iS5 with a diamond crystal. Spectra were recorded from 500 to 3800 cm^−1^ with 16 scans and a resolution of 4 cm^−1^, optical velocity of 0.4747, and aperture of 100 %.

The Thermo Scientific Nicolet iS5 FT-IR spectrometer used in this study maintains wavenumber accuracy through an internal He–Ne laser calibration system. Instrument performance and wavenumber precision were regularly validated using a standard polystyrene film according to the ASTM E1421 validation practices and European Pharmacopoeia (2.2.24) [18] guidelines, to ensure consistent wavenumber precision and signal quality during spectral acquisition (Thermo Fisher Scientific, 2016) [19]. All spectra were collected under stable environmental conditions (temperature 23±1 °C, controlled humidity) to minimize variation caused by atmospheric CO₂ and water vapor.

### 4.3 Spectral pre-processing

Baseline correction and normalization of the FTIR spectra were performed using the academic freeware SpectraGryph 1.2.16.1. The spectra were normalized to the most intense band within the fingerprint region (850–1850 cm⁻¹).

## Limitations

As shown in Table 2, there is considerable variation in sample size among species (e.g., five spectra for Common jasmine vs. 63 for Meadowsweet), which may affect the statistical robustness of results for species with fewer samples. This imbalance partly reflects limited material availability and the small number of producers for certain plants. Consequently, results for these species should be interpreted with caution.

## Ethics Statement

The authors confirm that they have read and comply with the ethical requirements for publication in Data in Brief. This work does not involve human subjects, animal experiments, or any data collected from social media platforms.

## CRediT Author Statement

**Una Lote Vītoliņa:** Writing – original draft preparation, Data curation, Visualization; **Jurģis Krūmiņš:** Writing – original draft preparation; **Renāte Teterovska:** Writing – review and editing, Resources; **Ance Bārzdiņa:** Writing – review un editing, Resources; **Baiba Mauriņa:** Writing – review and editing, Resources; **Dace Bandere:** Writing – review and editing, Supervision, Resources; **Agnese Brangule:** Supervision, Conceptualization, Methodology, Writing – review and editing.

## Data Availability

ZenodoLatvian Herbal Medicines Under the Infrared Lens: An FTIR-ATR Dataset (Original data)
